# Proteogenomic identification of Hepatitis B virus (HBV) genotype-specific HLA-I restricted peptides from HBV-positive patient liver tissues

**DOI:** 10.3389/fimmu.2022.1032716

**Published:** 2022-12-13

**Authors:** Mayank Srivastava, Richard Copin, Augustine Choy, Anbo Zhou, Olav Olsen, Sarah Wolf, Darshit Shah, Anna Rye-Weller, Lisa Chen, Newton Chan, Angel Coppola, Kathryn Lanza, Nicole Negron, Min Ni, Gurinder S. Atwal, Christos A. Kyratsous, William Olson, Robert Salzler

**Affiliations:** Regeneron Pharmaceuticals Inc., Tarrytown, NY, United States

**Keywords:** proteogenomics, Hepatitis B, hepatocellular carcinoma, immunopeptidomics, human leukocyte antigen (HLA)

## Abstract

The presentation of virus-derived peptides by HLA class I molecules on the surface of an infected cell and the recognition of these HLA-peptide complexes by, and subsequent activation of, CD8^+^ cytotoxic T cells provides an important mechanism for immune protection against viruses. Recent advances in proteogenomics have allowed researchers to discover a growing number of unique HLA-restricted viral peptides, resulting in a rapidly expanding repertoire of targets for immunotherapeutics (i.e. bispecific antibodies, engineered T-cell receptors (TCRs), chimeric antigen receptor T-cells (CAR-Ts)) to infected tissues. However, genomic variability between viral strains, such as Hepatitis-B virus (HBV), in combination with differences in patient HLA alleles, make it difficult to develop therapeutics against these targets. To address this challenge, we developed a novel proteogenomics approach for generating patient-specific databases that enable the identification of viral peptides based on the viral transcriptomes sequenced from individual patient liver samples. We also utilized DNA sequencing of patient samples to identify HLA genotypes and assist in target selection. Liver samples from 48 HBV infected patients, primarily from Asia, were examined to reconstruct patient-specific HBV genomes, identify regions within the human chromosomes targeted by HBV integrations and obtain a comprehensive view of HBV peptide epitopes using our HLA class-I (HLA-I) immunopeptidomics discovery platform. Two previously reported HLA associated HBV-derived peptides, HLA-A02 binder FLLTRILTI (S_194-202_) from the large surface antigen and HLA-A11 binder STLPETTVVRR (C_141-151_) from the capsid protein were validated by our discovery platform, but both were detected at very low frequencies. In addition, we identified and validated, using heavy peptide analogues, novel strain-specific HBV-HLA associated peptides, such as GSLPQEHIVQK (P_606-616_) and variants. Overall, our novel approach can guide the development of bispecific antibody, TCR-T, or CAR-T based therapeutics for the treatment of HBV-related HCC and inform vaccine development.

## Introduction

Chronic HBV infection affects ~250 million people worldwide and can result in serious complications including acute and chronic hepatitis, cirrhosis, and hepatocellular carcinoma (HCC) (1). Vaccination against HBV is effective at preventing infection, but there is currently no cure for patients chronically infected with the virus. Prolonged exposure of the liver to high viral loads results in persistent inflammation, which can stimulate fibrogenic and cirrhotic processes that can eventually lead to HCC. Additionally, the integration of HBV DNA fragments into the host genome can introduce genetic damage and chromosomal instability that can further contribute to the development of HCC (2). Globally, it is estimated that chronic HBV infections account for ~50% of primary liver cancers (3).

The persistence of HBV infection, or its control, is determined by the host immune response, which, following HBV evasion of the innate immune system, is primarily mediated by the adaptive immune system. Cytotoxic CD8+ T cells protect a host against intracellular pathogens and tumors (4). As such, cytotoxic CD8+ T cells play a pivotal role in defending against HBV infection, and are primarily responsible for clearing virus in acute infections (5), as well as preventing development of HCC. In cases of chronic HBV infection, HBV-specific CD8+ T cells are frequently undetectable, which is thought to result from the chronic exposure of the liver to high antigenic loads and tolerogenic hepatocytes (6).

Because of the critical role of CD8+ T cells in defending against viral infections, the cytotoxic T cell activity against infected cells can be utilized as an effective therapeutic approach. Cytotoxic T-cells mediate adaptive immunity by recognizing HLA-peptide complexes presented on the surface of infected cells *via* interactions with the T cell receptors (TCRs) expressed on their surface. These HLA-peptide complexes are utilized as targets for developing different immunotherapeutics including bispecific antibodies and CAR-Ts, but a lack of broadly expressed epitopes has made it difficult to implement these therapeutic strategies. Early attempts at identifying HBV-specific T cells typically utilize various T cell stimulation/proliferation assays (7, 8). However, the presence of these T cells does not necessarily mean that the peptide/HLA that they recognize is present HBV/HCC tumor cells. The most direct way to identify targetable HBV-HLA epitopes in HBV-HCC is to perform immunopeptidomics on these cells.

The emerging field of proteogenomics has been successfully applied to identify unique HLA-associated mutant peptides in cancer, characterizing HLA-presented epitopes that are expressed specifically by various tumors types (9–12). Previous work on identifying HBV-HLA epitopes in HCC has been performed, identifying several HBV peptides (13, 14). The greatest challenge to identifying these epitopes from HBV-HCC tissue is that HBV covalently closed circular DNA (cccDNA), the template for viral replication, is frequently absent from tumor cells. Instead, tumor cells contain fragments of HBV DNA that have been inserted into the host genome (15) that can be transcribed and, ultimately, result in the expression of viral antigens on the cell surface. This poses a challenge for identifying HBV epitopes as the exact regions of the HBV genome that are integrated can differ from patient to patient (16). Further complicating this effort is the existence of thousands of HBV strains distributed across 10 different HBV genotypes (genotype A to J) (17, 18), which differ from each other by at least 8% at the nucleotide level. These challenges emphasize the need for a new proteogenomic approach for comprehensive identification of HBV epitopes from patients. Here, we developed a novel proteogenomic approach to detect HLA-associated HBV peptides from HBV infected cells. We focused our efforts on characterizing the repertoire of HLA-I-HBV peptides that are expressed in the livers of infected patients, as this approach would likely provide the most accurate representation of HBV epitopes in the population. To achieve this, we performed bulk RNA sequencing of HBV-infected liver samples from Asian populations (19) to generate patient-specific HBV genome and construct associated-transciptome databases that were used to inform HLA-I-HBV peptide discovery from the same liver tissue. HLA-I associated peptides were purified from homogenized liver samples using immunoenrichment of HLA-I complexes and identified by subsequent mass spectrometric analysis (20). Our analysis identified previously reported HBV antigens derived from the large surface antigen and polymerase proteins as well as a novel HLA-A11 associated polymerase peptide. The polymerase peptide and its associated sequence variants collectively were found in a majority of our HLA-A11+ patients. We demonstrate that our proteogenomic discovery platform provides a new avenue for identifying patient specific HBV genome and associated-peptides as targets for anti-HBV related-HCC immunotherapy.

## Experimental section

### HBV-positive HCC tissue procurement

All patient tissues were purchased from Proteogenex or BioIVT in fresh frozen form. The patient details including age, sex and diagnostic tests are provided in **Supplementary Table S1**. Tissues were cryopreserved until sample preparation.

### RNA sequencing

Total RNA was extracted from human liver tissue using MagMAX kit (ThermoFisher). Strand-specific RNA-seq libraries were prepared from 1 µg RNA using KAPA stranded mRNA-Seq Kit (KAPA Biosystems). Twelve-cycle PCR was performed to amplify libraries. The amplified libraries were size-selected at 400~600 bp using PippinHT (Sage Science). Sequencing was performed on Illumina HiSeq^®^2500 (Illumina) by multiplexed paired-read run with 2X100 cycles.

### Mapping of patient liver RNA sequences to HBV reference genome

Bulk RNA-seq reads were aligned to reference genome ayw (NC_003977.2) using minimap2 (v2.17) (21). Alignments were then sorted by coordinates and quality controlled by samtools flagstats (v1.9) (22) and bedtools genomeCoverageBed (v2.17.0) (23) inspection. Duplicates were then marked and removed by the Picard toolkit (v2.18.2) (https://github.com/broadinstitute/picard).

### Workflow to reconstruct HBV genomes from patient liver RNAseq data

Paired-end Illumina RNA reads from each sample were *de novo* assembled into large contigs using megahit (24) (options: –min-count 3 –k-min 27 –k-max 127 –prune-level 2) and mapped using BLAST to HBV reference genome, ayw (NC_003977.2) to select HBV specific sequences. BLAST parameters for sequence comparisons included outfmt ‘7 std sgi stitle’; minimum E value = 0.001; cost to open a gap = 5; cost to extend a gap = 2; length of best perfect match = 11; reward for a nucleotide match = 2; reward for a nucleotide mis-match = -3. Contigs without BLAST matches were discarded as well as BLAST results with E values greater than 0.001, percentage identity below 79% or alignment length of less than 50 nucleotides. Overlapping contigs were merged using custom scripts and final sequence that covered the entire or partial length of the reference sequence with the highest identity was selected. The final sequence was mapped against the reference genome to extract and translate all coding sequences. Non-redundant protein sequences were then added to customized databases for peptide identification by mass-spectrometry.

### Workflow to identify integration sites from patient liver RNAseq data

Contigs matching the HBV reference genome (ayw) were extracted and mapped against human reference genome (GRCh38) using BLAST with parameters described above. Contigs matching both HBV and human (hybrid contigs) were collected for annotation. The HBV and human integration sites of the hybrid contigs were annotated against the genome features in HBV ayw Genbank file and human GRCh38 GTF file.

### HLA genotyping for patient samples

Sample Preparation and Sequencing: DNA sample quantity was determined by fluorescence (Life Technologies) and quality was assessed by running 25 ng of sample on a 1% pre-cast agarose gel (Life Technologies). Samples with high molecular weight gDNA with a majority of the DNA fragments greater than 20 kb and a concentration of no less than 10 ng/ul passed the quality assessment. The DNA samples were normalized to 10 ng/ul and 50 ng was used to amplify the full-length HLA amplicons. The targets were amplified in three pools of variant tolerant primers optimized for similar binding temperatures and PCR conditions with LA Taq DNA Polymerase (Takara Bio). The resulting amplicon pools were combined equimolarly as determined by automated capillary electrophoresis (Agilent) and fluorescence (Life Technologies). DNA libraries were prepared for Illumina-based sequencing with a custom NEB kit (New England Biolabs). The amplicons were enzymatically fragmented to a mean insert size of 250 bp and universal adapters were ligated onto the DNA fragments. Unique 10 base pair barcode sequences were added to the DNA fragments during PCR with NEBNext Ultra II Q5 Master Mix (New England Biolabs, M0544L) to facilitate highly multiplexed sequencing. The samples were pooled and sequenced using 150 base pair paired-end sequencing on an Illumina Nextseq 500.

Data Analysis: Upon completion of sequencing, raw data from each Illumina Nextseq run was gathered in local buffer storage and uploaded to a local high-performance computing platform for automated analysis. The FASTQ-formatted reads were converted from the BCL files and assigned to samples identified by specific barcodes using the bcl2fastq conversion software (Illumina Inc., San Diego, CA). All the reads in sample-specific FASTQ files were subjected to HLA typing analysis using an updated version of PHLAT program (25) with the reference sequences consisting of GRCh38 genomic sequences and HLA type reference sequences in the IPD-IMGT/HLA database v3.30.0 (26).

### Tissue lysis, and HLA affinity enrichment

The tissues were pulverized using SPEX SamplePrep Freezer/Mill Dual-Chamber Cryogenix Grinder (Cole-Parmer) in liquid nitrogen, and lysed in ice-cold lysis buffer (1% NP-40, 150 mM NaCl, 50 mM Tris-HCl pH 8.0 and 10 mM EDTA pH 8.0) supplemented with HALT protease and phosphatase inhibitors (Thermo Scientific, cat. 78442) on ice using sonication.

Anti-HLA Class I (W6/32) was conjugated to NHS-sepharose beads by overnight incubation in coupling buffer (0.2 M NaHCO_3_ + 0.5 M NaCl pH 8.3) in 4°C. Reaction was quenched with 0.1 M Tris-HCl pH 8.5, and beads were washed with the Tris-HCl solution and 0.1 M acetate buffer.

The pre-cleared tissue lysate was passed through a column packed with 1 ml of HLA Class-I beads bed under gravity. The column was subsequently washed with Seppro Dilution Buffer (Sigma, cat. S4199) and 20 mM Tris-HCl pH 8, and HLA-peptide complexes were eluted with 0.1 M glycine pH 2.7.

### Sample preparation for mass spectrometry

The glycine elute was loaded onto the C_18_ Sep-Pak (Waters, cat. WAT054960), followed by selective elution of peptides by 30% ACN/0.1% TFA. The peptides were further cleaned up by ziptip (C_18_ resin), and analyzed by nano-LC-MS/MS.

### LC-MS/MS

HLA peptides as above were loaded onto a nanoViper Acclaim PepMap100 C_18_ trap column (75 μm i.d. × 2 cm, 3 μm, 100 Å, Thermo) and were separated using a nanoViper Acclaim PepMap RSLC C_18_ column (75 μm i.d. × 25 cm, 2 μm, 100 Å, Thermo) heated to 40°C and retrofitted with a New Objective SilicaTip (7 cm) with a distal conductive coating at the inlet end of the emitter. The gradient was delivered by an EASY-nLC 1200 HPLC system (Thermo) at 300 nL/min. The following 120-minute elution gradient with mobile phase A (Water/0.1% formic acid) and B (80% Acetonitrile/0.1% formic acid) was used: 3% B at 3 min, linear to 35% B at 100 min, and linear to 45% B at 123 min. The peptides eluted from the column were ionized *via* Flex ion source at 1.9 kV and analyzed by the Thermo Fusion Lumos Tribrid mass spectrometer (Thermo) using Xcalibur 4.1.31.9 (Thermo). Data acquisition was performed in data-dependent mode, where survey scans were carried out in the high field Orbitrap analyzer (range of m/z 300-1500 at a resolution of 60,000) with the automatic gain control target of 4.0E5 and maximal ion fill time of 100 ms. The MS/MS analyses were performed by 1.2 m/z precursor ion isolation with the quadrupole, applying normalized HCD (higher-energy collisional dissociation) collision energy of 32%, and analysis of fragment ions in the Orbitrap at a resolution of 15,000. Dynamic exclusion window was set to 6 seconds, monoisotopic precursor selection (MIPS) to peptide, maximum injection time to 100 ms, and charge states unknown. +1-+4 charge states were included and the advanced peak determination was toggled on. For FAIMS-enabled experiments, the settings were identical except the FAIMS device was placed between the nanoelectrospray source and the mass spectrometer. FAIMS separations were performed with the following settings: inner and outer electrode temperature set to 100°C (except where noted), FAIMS carrier gas flow of 5.0 L/min, asymmetric waveform with DV −5000 V, entrance plate voltage 250 V, and CV settling time of 25 ms. The FAIMS carrier gas is N_2_, and the ion separation gap is 1.5 mm. The noted CVs were applied to the FAIMS electrodes. For external stepping or single CV experiments, the selected CV was applied to all scans throughout the analysis. For internal CV stepping experiments, each of the selected CVs was applied to sequential survey scans and MS/MS cycles (1 s); the MS/MS CV was always paired with the appropriate CV from the corresponding survey scan.

### Identification of HBV peptides from HLA-I immunopeptidomics of patient liver samples

All mass spectrometry raw files were searched against a consolidated database of human UniProtKB (*homo sapiens*) and HBV protein sequences, obtained from RNAseq of patient liver samples as described above, with PEAKS DB search engine (27). PEAKSX+ (PEAKS Studio 10.5, Bioinformatics Solutions Inc.) was used for *De novo*-assisted database search with precursor mass tolerance of 8 ppm, and fragment ion tolerance of 0.02 Da. Enzyme selectivity was set to none, and methionine oxidation as the only variable modification with two maximum allowed modifications per peptide. The search was performed with a 5% false-discovery rate (FDR) at peptide level, and peptides were further filtered based on -logP score of 20 (corresponding to 1% FDR).

### Peptide validation using synthetic heavy peptide analogues

The HLA-eluted peptides were mixed with synthetic peptides (50 fmol) containing heavy Leucine (^13^C(6)^15^N(1), +7.0172) and analyzed by LC-MS/MS. The identity of endogenous peptides was confirmed by comparing the elution and fragementation profile with the corresponding heavy peptides. Furthermore, the abundance of endogenous peptides was estimated based on the intensity of heavy and endogenous peptides, using Avogadro’s number. For this calculation, we assumed a 50% sample loss during the sample preparation, and 100 million cells as the sample amount.


$$\frac{\frac{Endogenous\;intensity}{Heavy\;Intensity}\times \;\left(6.023\;\times \;10^{23}\;copies/mol\right)\times \left(1\times 10^{-15}\;mol\right)}{\left(1\times 10^{8}\;cells\right)\times \;50\%}$$

## Results

### Generation of RNAseq databases from HBV infected patient samples

Comparative genomic analyses are often guided by a limited number of ‘reference’ genomes. As of August 2022, the National Center for Biotechnology and Information (NCBI) database contains a repository of more than 14,300 HBV genome sequences classified into at least ten distinct phylogenetic lineages (A to J https://www.ncbi.nlm.nih.gov/nuccore). While each lineage is represented by a single HBV genome, these reference sequences do not capture the full breadth of genomic diversity present in nature. This lack of coverage can negatively impact identification of new viral peptides as the sequence of patient isolates can greatly diverge from the reference genome sequence used for the comparative analysis.

**Figure 1:**
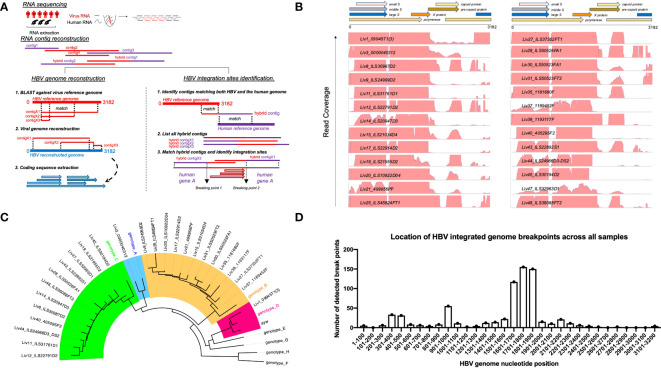
Approach to identify HBV specific signatures using bulk RNA sequencing of human liver samples. **(A)** *De-novo* sequencing strategy to reconstruct patient specific HBV genomes and identify HBV-integration sites within human chromosomes. RNA reads from patient samples are assembled into large contigs. Contigs fully matching HBV reference are used to reconstruct genomes from patient-infecting HBV strains. Specific coding sequences from each reconstructed genome are saved into a large patient-specific HBV database. In parallel, hybrid contigs matching both human and HBV references are filtered to identify site of integration within the human genomes using a homology sequence alignment approach. **(B)** Coverage of total RNA reads from bulk RNA sequencing of all human liver samples to HBV reference genome. **(C)** Phylogenetic relationship between reconstructed HBV genomes and reference HBV sequences representative of the eight HBV lineages (A–H). The tree is generated using a Neighbor-joining phylogenetic approach rooted on the mid-point. **(D)** Location of HBV integrated genome breakpoints across all samples. The RNAseq data revealed junctions where HBV sequences were integrated into human sequences. We noted the HBV sequence at these junctions and determined their location in the HBV genome, designated as “breakpoints”. The frequency of the genomic locations of these breakpoints across all studied samples are displayed here in 100 nucleotide intervals.

To circumvent this problem, we employed a novel RNAseq-based approach to generate a database of patient-specific HBV genome sequences directly reconstituted from the transcribed virus RNA sequences amplified from patient liver samples (**Figure 1A**). Total human RNA reads are first converted into large contigs using a *de-novo* assembly approach, and contigs with loose homology to HBV reference genomes are marked as HBV-specific sequences. HBV contigs are then grouped into two categories based on the percentage of the contig sequence covering the HBV reference. Fully-mapped contigs to HBV reference are re-arranged in a reference free fashion to assemble patient-specific HBV genomic sequences. Contigs with hybrid matches to the HBV reference are mapped against the human genome and the hybrid contigs matching both HBV and human sequences are selected. The patient-specific viral sequence is then used to extract and translate all HBV coding sequences (**Figure 1B**). Using this approach, we identified a total of 80 unique coding sequences (large S= 14, middle S= 14, small S= 16, pre-capsid= 6, capsid= 12, X= 16 and polymerase= 2) retrieved from virus isolates that are representative of four phylogenetic lineages (A, B, C, D) (**Figure 1C**). In accordance with the reported prevalence of HBV genotypes in Asian populations, HBV genotypes B and C were most commonly identified, having been found in 24 of the 48 samples (12 each). HBV genotypes A and D were detected in only one patient sample each (**Table 1**). RNA sequencing data for 18 of the samples had low HBV coverage, suggesting either an absence of HBV virus in these samples, possibly due to viral latency or clearance, or low HBV expression.

**Table 1:** The Patient sample ID, diagnosis from vendor, and HBV serological test results for all the patient samples analyzed, along with number of HBV unique peptides detected by mass spectrometry and HBV genotype identified from RNAseq results.

Sample ID	Diagnosis (from vendor)	HBV status from vendor	HBV Peptides by MS (5% FDR, 9-12 residues)	Number of HBV peptides detected by MS (5% FDR, 9-12 residues)	HBV Genotype from RNAseq	Virus reads
Liv1_09945T1(3)	liver cancer	HBV	Yes	4	D	11865
Liv2_0000032532	Cirrhosis	HBV	No	0	*low coverage*	13
Liv3_0000040312	Cirrhosis	HBV	Yes	3	A	5205
Liv4_0000031295	Cirrhosis	HBV	No	0	*low coverage*	68
Liv5_0000049186	Chronic inflammation	HBV	Yes	12	poor QC	0
Liv6_0000049219	Hepatitis	HBV	Yes	1	poor QC	0
Liv7_ILS37316FT2	HCC	HBV	No	0	*low coverage*	26
Liv8_ILS30987D2	HCC	HBV	Yes	6	C	14633
Liv9_ILS24999D2	HCC	HBV	Yes	5	B	9423
Liv10_ILS39940FT1	HCC	HBV	Yes	4	poor QC	0
Liv11_ILS31761D1	HCC	HBV	Yes	3	C	9935
Liv12_ILS22791D2	HCC	HBV	Yes	7	C	26154
Liv13_ILS23304D2	HCC	HBV	No	0	*low coverage*	31
Liv14_ILS20947D3	HCC	HBV	Yes	2	C	2070
Liv15_ILS21034D4	HCC	HBV	Yes	4	B	5369
Liv16_ILS42208FT1	HCC	HBV	Yes	4	poor QC	0
Liv17_ILS22914D2	HCC	HBV	Yes	1	B	12853
Liv18_ILS20954D2	HCC	HBV	No	0	*low coverage*	23
Liv19_ILS21955D2	HCC	HBV	Yes	1	C	3909
Liv20_ILS10922D04	HCC	HBV	Yes	11	B	26200
Liv21_499956PF	HCC	HBV	Yes	2	B	9010
Liv22_524614VF	HCC	HBV	No	0	*low coverage*	30
Liv23_ILS35989FT2	HCC	HBV	No	0	*low coverage*	4
Liv24_ILS42195FT1	HCC	HBV	No	0	*low coverage*	6
Liv25_ILS45824FT1	HCC	HBV	Yes	4	B	43486
Liv26_ILS36725FT2	HCC	HBV	No	0	*low coverage*	6
Liv27_ILS37352FT1	HCC	HBV	Yes	2	B	15594
Liv28_ILS50526FA1	Hepatitis, chronic (Diseased)	HBV	Yes	4	C	22294
Liv29_ILS50526FT2	HCC	HBV	No	0	*low coverage*	30
Liv30_ILS50523FA1	Cirrhosis (Diseased)	HBV	Yes	6	B	18692
Liv31_ILS50523FT2	HCC	HBV	Yes	5	B	14716
Liv32_1176935F	HCC	HBV	No	0	*low coverage*	1
Liv33_1176559F	HCC	HBV	No	0	*low coverage*	18
Liv34_1182970F	HCC	HBV	No	0	*low coverage*	88
Liv35_1181680F	HCC	HBV	Yes	4	B	9563
Liv36_1182812F	HCC	HBV	No	0	*low coverage*	990
Liv37_1189452F	HCC	HBV	Yes	2	B	1468
Liv38_1193117F	HCC	HBV	Yes	9	B	57394
Liv39_1192879F	HCC	HBV	No	0	*low coverage*	25
Liv40_405295F2	HCC	HBV	Yes	1	C	52627
Liv41_ILS32961D2	HCC	HBV	No	0	*low coverage*	1
Liv42_ILS33989D2	HCC and Cholangiocarcinoma	HBV	No	0	*low coverage*	17
Liv43_ILS22892S1	HCC	HBV	Yes	2	C	9174
Liv44_ILS24966D3-DS2	HCC and Cholangiocarcinoma	HBV	Yes	3	C	2980
Liv45_ILS30194D2	HCC	HBV	Yes	3	C	4486
Liv46_ILS32365D1	HCC	HBV	No	0	*low coverage*	39
Liv47_ILS32963D1	HCC	HBV	Yes	1	C	675
Liv48_ILS36088FT2	HCC	HBV	Yes	1	C	16030

Read mapping analysis showed that the depth of coverage of virus reads was high and consistent in the genomic region encoding the S and X proteins, while coverage for the rest of the genome contained significant gaps (**Figure 1B**). Additionally, these reads often terminate in the middle of an HBV gene. These gaps and partial reads suggest that the reads could be coming from RNA transcribed from partially integrated HBV DNA sequences instead of cccDNA. Of note, the N-terminal region of the genome-spanning polymerase gene was poorly covered, whereas the C-terminal portion of the polymerase gene was well detected. For the majority of HBV+ samples, we also identified hybrid contigs combining both HBV and human sequences. These sequences are expected to result from integration of HBV DNA into the coding sequence of a human gene. The genomic location of the HBV sequence in these junctions is referred to as “breakpoints”. We mapped the HBV breakpoints found in RNAseq reads with HBV/human junctions and found that the majority of HBV breakpoints occurred between nucleotide positions 1601-1900 in the HBV genome (**Figure 1D**) while no clear pattern was observed for the host genome (**Supplementary Table S2**). This data is consistent with a previous report by Yang et al (28), suggesting that at least some of the detected HBV sequences are coming from integrated DNA and not cccDNA. The reconstitution of HBV genome and the HBV sequences from patient liver samples were utilized to discover HBV peptides presented on the surface of HCC tumors.

### Characterization of the immunopeptidome of HBV-infected patient samples

**Figure 2:**
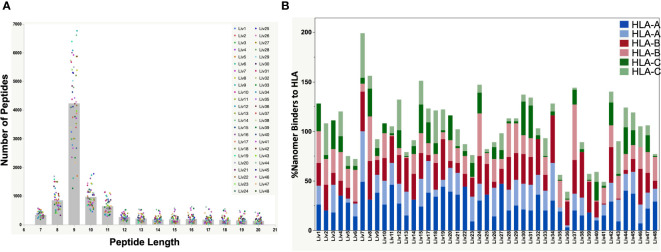
**(A)** Peptide length distributions by mass spectrometry for all HBV-positive patient liver tissues by serology. The raw files were searched with PEAKSX with 5% FDR at peptide level. A vast majority of 9-mers (peptides with 9 amino acid residues) were detected from the HLA class-I enrichment from a high percent of patient liver samples. **(B)** Plot showing the %Binders to HLA-A, HLA-B and HLA-C alleles from identified 9-mer peptides by mass spectrometry. The HLA alleles were identified from DNA sequencing of each patient sample. A vast majority of detected nonamer peptides by mass spectrometry were predicted binders to the patient HLA alleles predicted by NetMHC pan 4.0. The combined percent binders to the three HLA alleles was over 100% for some of the samples due to promiscuity in peptide binding to HLA as predicted by NetMHC (**Supplementary Table S3**).

HLA Class-I complexes were immunopurified from homogenized hepatocellular carcinoma (HCC) liver tissue samples from 48 HBV seropositive patients (Proteogenex and BioIVT, **Table 1**, **Supplementary Table S1**) using an anti-HLA Class-I antibody (W6/32). Western blotting of liver lysates, flow throughs, and enrichment elutions demonstrated a near complete capture of HLA-I by W6/32 linked sepharose beads (**Supplementary Figure S1**). Following elution of the complexes, HLA-I bound peptides were identified using an Orbitrap Fusion Lumos mass spectrometer coupled to a nanoLC system. The raw mass spectrometry (MS) data files were searched against a combined database of human UniProt and HBV sequences derived from the RNA HBV reads from each patient sample. On average, approximately 8,500 total peptides were detected from liver samples from variably aged patients (**Supplementary Figure S2**). The distribution of peptide lengths isolated from the majority of liver tissue samples was consistent with those expected for Class I HLA enrichment, with peptides nine residues in length (9-mers) being the most abundant, followed by peptides ten (10-mers) and eleven (11-mers) residues in length (**Figure 2A**). Peptides with nine to twelve residues constituted over 50% of the total peptides isolated (**Supplementary Figure S2**).

### High percentage of identified peptides are predicted to bind HLA using NetMHC pan 4.0 analysis

The polymorphic nature of HLA-I has a profound effect on the epitopes presented on the tissue surface. Hence, DNA sequencing of liver tissue samples was performed to determine the HLA genotype (**Table 2**), which was used to predict the binding of detected peptides by NetMHC pan 4.0 (29). We found that a very high percentage (50 – 100% for most of the samples) of the detected 9-mer peptides were predicted binders of the identified HLA-I in the corresponding patient samples (**Figure 2B**; **Supplementary Table S3**). The percent of nonamers that bound to the patient HLAs exceeded 100% for some samples, owing to the promiscuity in peptide binding predicted by NetMHC Pan 4.0.

**Table 2:** HLA class I alleles identified for all HBV-infected patient samples from DNA sequencing.

	HLA-A				HLA-B				HLA-C			
Sample ID	Allele 1		Allele 2		Allele 1		Allele 2		Allele 1		Allele 2	
Liv1_09945T1(3)	*02	01	*24	02	*38	01	*39	01	*12	03	*12	03
Liv2_0000032532	*03	01	*03	01	*15	16	*53	01	*04	01	*14	02
Liv3_0000040312	*11	01	*24	02	*27	06	*38	02	*07	02	*07	02
Liv4_0000031295	*24	02	*24	02	*13	01	*40	01	*03	04	*07	02
Liv5_0000049186	*02	01	*03	01	*07	02	*38	01	*04	01	*07	02
Liv6_0000049219	*11	01	*30	01	*13	02	*40	01	*06	02	*07	02
Liv7_ILS37316FT2	*02	03	*02	06	*15	25	*55	02	*07	02	*12	03
Liv8_ILS30987D2	*11	01	*11	01	*15	02	*35	01	*03	03	*08	01
Liv9_ILS24999D2	*02	03	*33	03	*38	02	*48	03	*07	02	*08	01
Liv10_ILS39940FT1	*11	01	*30	01	*07	02	*38	02	*07	02	*07	02
Liv11_ILS31761D1	*02	03	*11	01	*15	02	*46	01	*01	02	*08	01
Liv12_ILS22791D2	*02	01	*11	01	*39	01	*51	01	*07	02	*14	02
Liv13_ILS23304D2	*11	01	*24	02	*35	05	*46	01	*01	02	*04	01
Liv14_ILS20947D3	*11	01	*11	01	*15	02	*38	02	*07	02	*08	01
Liv15_ILS21034D4	*11	01	*33	03	*15	02	*58	01	*03	02	*14	02
Liv16_ILS42208FT1	HLA genotype couldn’t be identified											
Liv17_ILS22914D2	*11	01	*31	01	*13	01	*51	02	*03	04	*15	02
Liv18_ILS20954D2	*02	01	*74	02	*15	02	*51	01	*08	01	*14	02
Liv19_ILS21955D2	*02	03	*31	01	*15	25	*51	01	*04	03	*16	02
Liv20_ILS10922D04	*02	264	*33	03	*18	02	*58	01	*03	02	*07	04
Liv21_499956PF	*02	1	*11	1	*13	1	*55	2	*01	2	*03	4
Liv22_524614VF	*11	1	*11	1	*18	2	*38	2	*04	3	*07	2
Liv23_ILS35989FT2	*02	7	*11	1	*38	2	*46	1	*01	12	*07	2
Liv24_ILS42195FT1	*11	1	*29	1	*07	5	*35	1	*03	3	*15	5
Liv25_ILS45824FT1	*24	2	*24	2	*40	1	*54	1	*01	2	*04	3
Liv26_ILS36725FT2	*01	1	*33	3	*13	1	*57	1	*03	4	*06	2
Liv27_ILS37352FT1	*11	1	*11	1	*38	2	*51	1	*07	2	*14	2
Liv28_ILS50526FA1	*11	1	*33	3	*15	25	*35	5	*04	1	*04	3
Liv29_ILS50526FT2	*11	1	*33	3	*15	25	*35	5	*04	1	*04	3
Liv30_ILS50523FA1	*11		*33	3	*15	2	*58	1	*03	2	*08	1
Liv31_ILS50523FT2	*11		*33	3	*15	2	*58	1	*03	2	*08	1
Liv32_1176935F	*02	6	*11	1	*15	2	*15	2	*07	2	*08	1
Liv33_1176559F	*24	2	*26	1	*38	2	*54	1	*01	2	*07	2
Liv34_1182970F	*11	1	*29	1	*15	2	*15	2	*04	3	*08	1
Liv35_1181680F	*11	1	*33	3	*44	3	*56	4	*07	6	*08	1
Liv36_1182812F	*11	1	*26	1	*44	3	*56	4	*07	6	*08	1
Liv37_1189452F	*11	1	*24	2	*15	12	*15	25	*03	3	*04	3
Liv38_1193117F	*11	1	*24	3	*15	2	*15	2	*04	3	*08	1
Liv39_1192879F	*11	1	*11	1	*15	2	*46	1	*01	2	*08	1
Liv40_405295F2	*02	7	*02	65	*38	2	*46	1	*01	12	*07	2
Liv41_ILS32961D2	*01	1	*02	7	*46	1	*57	1	*01	2	*06	2
Liv42_ILS33989D2	*02	1	*24	2	*15	2	*15	12	*03	3	*08	1
Liv43_ILS22892S1	*02	7	*33	3	*46	1	*58	1	*01	2	*03	2
Liv44_ILS24966D3-DS2	*02	3	*33	3	*13	1	*58	1	*03	2	*03	4
Liv45_ILS30194D2	*02	3	*24	2	*15	12	*40	1	*03	3	*07	2
Liv46_ILS32365D1	*02	7	*24	3	*15	1	*15	12	*01	3	*03	3
Liv47_ILS32963D1	*11	1	*31	1	*51	1	*51	2	*14	2	*15	2
Liv48_ILS36088FT2	*02	3	*11	1	*15	21	*56	4	*01	2	*04	3

Symbol * is the separator and it generally is associated with the first two digits (out of 4 digits) in HLA allele. The first two digits define the HLA type.

We next performed a motif analysis of detected peptides to identify overrepresented residues at different peptide positions using Seq2Logo (30) (**Supplementary Figure S3**). As expected, anchor residues at position 2 and 9, which specify HLA-I binding, were significantly overrepresented in all the tissue samples, with residue identities corresponding to the HLA-I genotype present. For example, hydrophobic anchor residues (i.e. Leu and Val) characteristic of HLA-A02 binders were enriched at positions 2 and 9 of peptides isolated from HLA-A02+ patient samples. Together, the high percentage of predicted HLA binders and the overrepresentation of characteristic anchor residues in isolated peptides provide a high degree of confidence in the HLA-I enrichment and peptide identifications.

### Identification of unique HLA-associated HBV peptides using HBV genotype-specific databases

**Figure 3:**
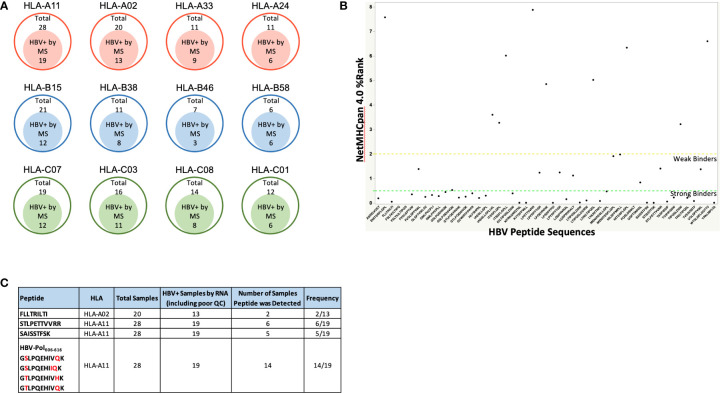
**(A)** The four most frequent HLA alleles from HLA-A, HLA-B and HLA-C among the patient samples analyzed, along with the number of samples that were HBV positive by Mass Spectrometry (HBV peptides detected). As expected, a high percent patient samples were HLA-02 and HLA-A11 samples due to Asian population being the source of the samples procured. **(B)** HBV peptide sequences identified from immunopeptidomics of patient liver samples, and their predicted binding to patient HLA alleles predicted by NetMHCpan 4.0. Strong binders are ranked below 0.5 and weak binders between 0.5 – 2, according to NetMHC prediction. For the HBV peptides with predicted binding to more than one alleles, the affinity rank for the strongest binding is shown. Refer to **Supplementary Table S4** for the complete list of HBV peptides along with their binding affinities to the patient HLA alleles predicted by NetMHCpan 4.0. **(C)**. Target HBV peptides and their frequencies of being detected in HBV+ samples. Refer to **Supplementary Table S5** for the patient samples in which these peptides were detected.

We reasoned that the number of HLA-I-presented HBV peptides would depend on the degree of HBV infection, proteasomal processing of antigen, and tissue sample quality. Indeed, the number of HBV specific peptide sequences identified from infected livers varied between samples and generally showed low sequence coverage of HBV proteins (**Table 1**; **Figure 1B**). In total, we identified 54 unique HBV peptides with lengths of nine to twelve residues using a PEAKS ion cut off score of 20 (**Table 3**). These peptides were detected from patient samples with diverse HLA genotype (**Figure 3A**). The most frequent HLA allele in the patient samples analyzed was HLA-A11 followed by HLA-B15 and HLA-A02. A majority of the viral peptides detected were NetMHC pan 4.0 predicted binders of the HLA-I allele present in the corresponding patient sample (**Figure 3B**; **Supplementary Table S4**). The large surface antigen protein was the source of a few strong HLA-A02 predicted binders (NetMHC pan 4.0) that were identified: GLSPTVWLSV (S_348-357_), GMLPVCPLL (S_276-284_), FLLTRILTI (S_194-202_) and GMLPVCPLI (S_276-284_), as well as a weak binder NILSPFMPLL (S_370-379_) and were all found at a relatively low frequencies (**Table 3**). In contrast, some HLA-A11 associated peptides: STLPETTVVRR (C_141-151_) from capsid protein and SAISSTFSK from polymerase were found at relatively higher frequencies (25-30%) (**Figure 3C**). Notably, we detected novel HLA-A11 restricted HBV-genotype specific polymerase peptides GSLPQEHIVQK (P_606-616_) and GSLPQEHIIQK (P_606-616_) from genotype B, and GTLPQEHIVHK (P_606-616_) and GTLPQEHIVQK (P_606-616_) from genotype C. The former peptide variant is predicted to be a weak HLA-A11 binder, while the latter three peptides are predicted to be strong binders of HLA-A11 by NetMHC pan 4.0 (**Figure 3B**). More importantly, this polymerase peptide and its variants (GSLPQEHIVQK, GSLPQEHIIQK, GTLPQEHIVHK and GTLPQEHIVQK) have a combined frequency of ~74% (14 out of 19 samples) in HLA-A11 tissue samples (**Figure 3C**, **Supplementary Table S5**). Notably, no HBV peptides were detected from any of the 18 tissues from which RNA sequencing had no or low HBV coverage, further validating our proteogenomic approach and highlighting a good correlation between the two complementary methods.

**Table 3:** HBV peptides and proteins detected by search against patient-specific databases.

Sample ID	HBV Peptides (5% FDR)	IonScore (-logP)	Length	m.z	HBV Protein
Liv1_09945T1(3)	LTIPQSLDSW	43	10	580.302	S
	TRILTIPQSL	33	10	571.3472	S
	FVGLSPTVWL	20	10	559.8152	S
	MENITSGFLGPL	46	12	639.8233	S
Liv2_0000032532	Not Detected				
Liv3_0000040312	LTIPQSLDSW	33	10	580.303	S
	TRILTIPQSL	22	10	571.3493	S
	MENITSGFLGPL	37	12	639.823	S
Liv4_0000031295	Not Detected				
Liv5_0000049186	VGLSPTVWL	39	9	971.5576	S
	FVGLSPTVW	38	9	1005.5411	S
	ITSGFLGPL	36	9	904.514	S
	LTIPQSLDSW	48	10	1159.6012	S
	NITSGFLGPL	41	10	1018.5593	S
	TRILTIPQSL	40	10	571.3502	S
	FVGLSPTVWL	33	10	559.8156	S
	ITSGFLGPLL	29	10	509.3024	S
	LTRILTIPQSL	43	11	627.8931	S
	ENITSGFLGPL	32	11	574.3026	S
	WTSLNFLGGTTV	47	12	648.3353	S
	MENITSGFLGPL	32	12	1278.6306	S
Liv6_0000049219	TRILTIPQSL	29	10	571.3505	S
Liv7_ILS37316FT2	Not Detected				
Liv8_ILS30987D2	HLYSHPIIL	40	9	546.8131	Polymerase
	LPQEHIVHK	34	9	367.545	Polymerase
	LPETTVVRR	30	9	535.8181	Polymerase
	TASPISSIF	31	9	922.4841	S
	GTLPQEHIVHK	55	11	629.8475	Polymerase
	STLPETTVVRR	32	11	420.2415	Polymerase
Liv9_ILS24999D2	IASGLLGPL	25	9	840.5204	S
	FVGLSPTVWL	28	10	559.8152	S
	NILSPFMPLL	23	10	572.8248	S
	MENIASGLLGPL	44	12	607.8264	S
	IASGLLGPLLVL	31	12	583.3806	S
Liv10_ILS39940FT1	SAISSTFSK	29	9	464.2422	S
	KPRKGMGTNL	35	10	551.3126	
	GSLPQEHIVQK	47	11	618.3405	Polymerase
	STLPETTVVRR	27	11	420.241	C
Liv11_ILS31761D1	QSPTSNHSL	40	9	970.4584	S
	GLSPTVWLSV	31	10	529.7979	S
	GTLPQEHIVQK	50	11	625.3479	Polymerase
Liv12_ILS22791D2	LPSDFFPSI	22	9	1022.5152	C
	GMLPVCPLL	34	9	942.5134	S
	FLLTRILTI	25	9	545.3545	S
	GMLPVCPLI	34	9	942.5134	S
	TRILTIPQSL	32	10	571.3494	S
	GTLPQEHIVQK	54	11	625.3477	Polymerase
	STLPETTVVRR	30	11	629.8578	Polymerase
Liv13_ILS23304D2	Not Detected				
Liv14_ILS20947D3	GTLPQEHIVHK	42	11	420.2349	Polymerase
	STLPETTVVRR	33	11	420.2417	Polymerase
Liv15_ILS21034D4	TVSAISSTF	32	9	912.4692	S
	TIPQSLDSW	21	9	523.7634	S
	GSLPQEHIVQK	36	11	618.3413	Polymerase
	KILTIPQSLDSW	59	12	700.8957	S
Liv16_ILS42208FT1	TASAISSTF	35	9	884.4364	S
	IPIPSSWAF	38	9	1017.5414	S
	LPYRPTTGR	42	9	530.7983	Polymerase
	FSLTKILTIPQ	34	11	630.8829	S
Liv17_ILS22914D2	GSLPQEHIVQK	44	11	618.3427	Polymerase
Liv18_ILS20954D2	Not Detected				
Liv19_ILS21955D2	GVWIRTPPAYR	34	11	439.2469	C
Liv20_ILS10922D04	PQSLDSWLT	24	9	523.7632	S
	LPYRPTTGR	24	9	354.2018	Polymerase
	LTIPQSLDSW	45	10	580.3043	S
	NILSPFMPLL	40	10	572.8265	S
	FVGLSPTVWL	32	10	559.8168	S
	FSLTKILTIPQ	39	11	630.8831	S
	LTIPQSLDSWW	23	11	673.3439	S
	KILTIPQSLDSW	47	12	700.8918	S
	MENIASGLLGPL	43	12	607.8276	S
	IASGLLGPLLVL	42	12	583.3824	S
	FSLTKILTIPQS	33	12	674.3987	S
Liv21_499956PF	GSLPQEHIVQK	19	11	412.5624	Polymerase
	SAISSTFSK	35	9	464.242	S
Liv22_524614VF	Not Detected				
Liv23_ILS35989FT2	Not Detected				
Liv24_ILS42195FT1	Not Detected				
Liv25_ILS45824FT1	KYTSFPWLL	37	9	577.8159	Polymerase
	YPALMPLYA	27	9	519.7706	Polymerase
	LYAAVTNFL	23	9	506.2791	Polymerase
	MENIASGLLGPL	34	12	607.8266	S
Liv26_ILS36725FT2	Not Detected				
Liv27_ILS37352FT1	GSLPQEHIVQK	41	11	412.5654	Polymerase
	SAISSTFSK	35	9	464.2455	S
Liv28_ILS50526FA1	STLPETTVVRR	33	11	420.2422	Polymerase
	LPFRPTTGR	27	9	348.8699	Polymerase
	ASRELVVSY	23	9	512.2773	C
	GTLPQEHIVHK	37	11	420.2355	Polymerase
Liv29_ILS50526FT2	Not Detected				
Liv30_ILS50523FA1	MENIASGLLGPL	38	12	607.8255	S
	LTIPQSLDSW	37	10	580.3024	S
	SAISSTFSK	33	9	464.2425	S
	VGLSPTVWL	33	9	486.2813	S
	FVGLSPTVWL	32	10	559.8156	S
	FVGLSPTVW	27	9	503.273	S
Liv31_ILS50523FT2	MENIASGLLGPL	36	12	607.8255	S
	SAISSTFSK	35	9	464.2427	S
	LTIPQSLDSW	34	10	580.3034	S
	TCIPIPSSW	31	9	502.2487	S
	FVGLSPTVWL	25	10	559.8158	S
Liv32_1176935F	Not Detected				
Liv33_1176559F	Not Detected				
Liv34_1182970F	Not Detected				
Liv35_1181680F	STISSTFSK	31	9	479.2466	S
	FVGLSPTVWL	30	10	559.8156	S
	GSLPQEHIVQK	23	11	412.5617	Polymerase
	MENIASGLLGPL	26	12	607.8254	S
Liv36_1182812F	Not Detected				
Liv37_1189452F	FVGLSPTVWL	24	10	559.8156	S
	MENIASGLLGPL	23	12	607.8256	S
Liv38_1193117F	LQDPRVRAL	35	9	534.3187	S
	FVGLSPTVW	25	9	503.2733	S
	HLYSHPIIL	36	9	546.8134	S
	ASRELVVSY	30	9	512.2762	C
	LQDPRVRALY	32	10	410.9025	S
	FVGLSPTVWL	31	10	559.815	S
	STLPETTVVRR	38	11	420.2418	Polymerase
	GSLPQEHIIQK	35	11	417.2343	Polymerase
	MENIASGLLGPL	39	12	607.826	S
Liv39_1192879F	Not Detected				
Liv40_405295F2	FVGLSPTVWL	29	10	559.8152	Polymerase
Liv41_ILS32961D2	Not Detected				
Liv42_ILS33989D2	Not Detected				
Liv43_ILS22892S1	LTIPQSLDSW	30	10	580.3041	S
	FVGLSPTVWL	24	10	559.8157	S
Liv44_ILS24966D3-DS2	FVGLSPTVW	29	9	503.2723	S
	LTIPQSLDSW	36	10	580.3026	S
	FVGLSPTVWL	31	10	559.8146	S
Liv45_ILS30194D2	FLLTRILTI	23	9	545.354	S
	TASPISSIF	21	9	461.7469	S
	TASPLSSIF	21	9	461.7469	S
Liv46_ILS32365D1	Not Detected				
Liv47_ILS32963D1	GTLPQEHIVHK	38	11	420.2349	Polymerase
Liv48_ILS36088FT2	GTLPQEHIVHK	42	11	420.2341	Polymerase

Peptide GSLPQEHIVQK, detected once with -logP of 19 was considered as a real identification (highlighted in red for sample Liv21_499956PF). For all other peptides, a score cut-off of 20 was used. The Mass Spectrometer raw files were searched against a consolidated database of human UniProtKB and HBV protein sequences, obtained from RNAseq of patient liver samples using PEAKSX+. Peptides were initially filtered by 5% False-Discovery Rate (FDR) followed by -logP of 20 as minimum cut-off score. Decoy peptides were removed. Only peptides of length 9 – 12 are shown in the table.

**Figure 4:**
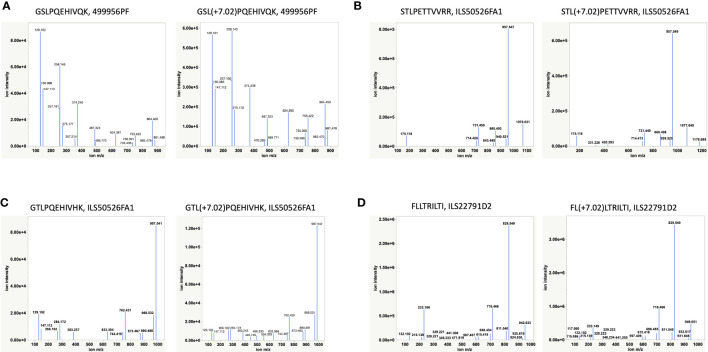
Validation of HBV peptides by heavy analogues (**Supplementary Table S6**). **(A, C)** Validation of two novel Polymerase variants. **(B, D)** Validation of two previously reported peptides by heavy peptide analogues. Synthetic heavy peptides with sequences analogous to target HBV peptides and a modified heavy leucine (Thermo), 50 fmol, were spiked-in into the HLA-eluted peptides and separated on the nano-LC connected online to the Mass Spectrometer. The MS raw files were searched against the combined database of human Uniprot and HBV with heavy leucine (+7.02) as a variable modification using PEAKS X. The fragmentation pattern (b- and y-ions) of HBV peptides and their heavy analogues along with their co-elution and identification in the same MS1 scan were used for peptide validation.

Having discovered novel HBV Polymerase variant peptides, which are absent in Uniprot and other public databases, for the first time utilizing RNAseq of patient samples, we next aimed to validate the peptide identities using synthetic peptide analogues. HBV peptides predicted to bind HLA-A02 and HLA-A11 by NetMHC, including FLLTRILTI (S_194-202_), STLPETTVVRR (C_141-151_), GSLPQEHIVQK (P_606-616_) and GTLPQEHIVHK (P_606-616_), were validated by retention time alignment and matching the fragmentation patterns of peptides with their synthetic heavy versions (**Figure 4** and **Supplementary Table S6**). The synthetic peptides used for this analysis differ from the HBV peptides by a heavy Leucine (^13^C(6)^15^N(1), +7.0172) and were mixed with HLA-eluted peptides prior to LC-MS/MS analysis. All peptides tested eluted at the same retention time and in the same MS1 scan as their heavy analogues in the LC-MS/MS run (**Supplementary Figure S4**) and also exhibited a similar fragmentation profile. The extracted ion chromatogram was also utilized to estimate the expression level or the copy number of some of the target peptides, including the polymerase peptide variants (copy number of ~100 – 500/cell) from the intensity of corresponding heavy peptide analogue parent ions. Even at relatively low surface presentation, we could differentiate between the polymerase peptide P_606-616_ variants based on the fragment ions (**Figure 4**). These HLA-I restricted HBV peptides provide novel targets for developing a potential immunotherapy.

## Discussion

T cell based therapeutics are a promising treatment for HBV-HCC. As such, the identification of appropriate peptide targets for these T cell therapeutics is critically important. Here we report the identification of HBV peptides from HBV+ HCC patient samples. We first reconstructed the specific HBV genome present in each patient sample using RNAseq. Then using these patient-specific sequences, we were able to analyze the LC-MS/MS data from each patient sample using an appropriate reference sequence that would account for any patient specific mutations. Some of the identified peptides have been previously reported (i.e. STLPETTVVRR (C_141-151_)) (31), while others are novel. We detected 45 previously unreported peptides out of 54 total identified HLA-I restricted HBV peptides (**Supplementary Figure S5**) (32). Of particular interest, we were able to identify a novel peptide GSLPQEHIVQK (POL_606-616_) and its variants from HBV polymerase. This peptide is a predicted HLA-A11 binder and is the most frequent HBV epitope found, appearing (together with its variants) in 74% of HLA-A11+ patient samples.

The reconstruction of the HBV genomes present in each sample also provides valuable insight to what regions of the HBV genome are integrated into hepatocytes during HCC development. We discovered that the region of the genome encompassing the small surface antigen protein, X protein, and the latter half of the polymerase protein was frequently detected. Conversely, the region encompassing the capsid protein and the first half of the polymerase protein was either not detected or only partially detected. This is in agreement with previous reports of integration of HBV genome fragments rather than the entire genome. Understanding what is frequently integrated is important to choosing a peptide target for T cell based therapeutics as a peptide that falls in a region of the HBV genome that is not commonly integrated into HCC cells would not be ideal.

Together, the combined use of the RNAseq data and LC/MS-MS data reported here demonstrate a sound approach to finding and choosing appropriate peptide targets for T cell based therapeutics. The ideal peptide target would be highly abundant on the surface of cells, found commonly across patients, conserved across multiple HBV genotypes, and fall in a region of the HBV genome that is commonly integrated in HCC. Using the methods reported here, we identified a peptide that meets some of these criteria. The POL_606-616_ peptide was frequently found in the HLA-A11 patient samples and does fall in the HBV genome region that is commonly integrated. Its conservation across HBV genotypes is not ideal as there is usually 2-3 amino acid changes across the different variants detected. However, it may be possible to design T cell based therapeutics around the regions of this peptide that are conserved. As we use this approach to examine more patient samples representing different HLA alleles, we may be able to find other peptides that fit more of these criteria.

## Data availability statement

The mass spectrometry immunopeptidomics data have been deposited to the ProteomeXchange Consortium via the PRIDE (33) partner repository with the dataset identifier PXD037270.

## Author contributions

MS, RC, AuC, OO, GA, CK, WO, and RS conceptualized the study. MS performed HLA immunoaffinity enrichment from patient samples, prepared samples for Mass Spectrometry, and analyzed data. RC conceptualized the strategy to identify HBV reads from bulk RNA sequencing of patient liver samples, and reconstruct HBV genomes. RC, AZ, and AuC performed read mapping analysis and also identification of integration sites. SW, NC, AnC, KL, NN, and MN performed DNA and RNA sequencing and HLA genotyping. OO, GA, CK, WO, and RS analyzed data and supervised the project. MS, RC, and AuC drafted the manuscript with contribution from OO and editing by WO and RS. All authors contributed to the article and approved the submitted version.
