# Supplementary material for: Proteogenomic identification of Hepatitis B virus (HBV) genotype-specific HLA-I restricted peptides from HBV-positive patient liver tissues
Source: Front Immunol. 2022 Dec 13;13:1032716. doi: 10.3389/fimmu.2022.1032716 (PMC9793402; doi:10.3389/fimmu.2022.1032716)
Supplement: Supplementary file 1 [file Presentation_1.pptx]

## Slide 1
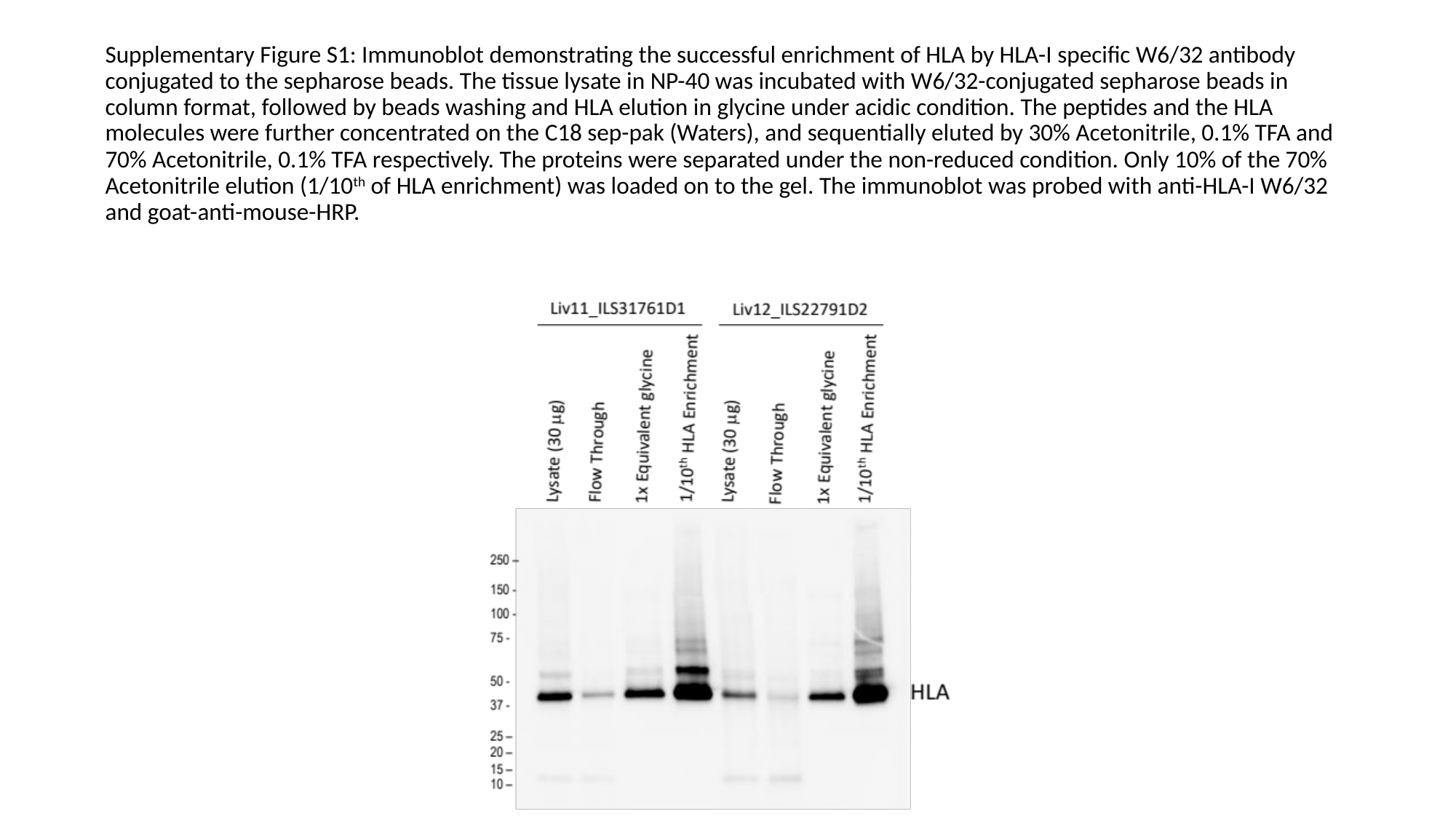

Supplementary Figure S1: Immunoblot demonstrating the successful enrichment of HLA by HLA-I specific W6/32 antibody conjugated to the sepharose beads. The tissue lysate in NP-40 was incubated with W6/32-conjugated sepharose beads in column format, followed by beads washing and HLA elution in glycine under acidic condition. The peptides and the HLA molecules were further concentrated on the C18 sep-pak (Waters), and sequentially eluted by 30% Acetonitrile, 0.1% TFA and 70% Acetonitrile, 0.1% TFA respectively. The proteins were separated under the non-reduced condition. Only 10% of the 70% Acetonitrile elution (1/10th of HLA enrichment) was loaded on to the gel. The immunoblot was probed with anti-HLA-I W6/32 and goat-anti-mouse-HRP.

## Slide 2
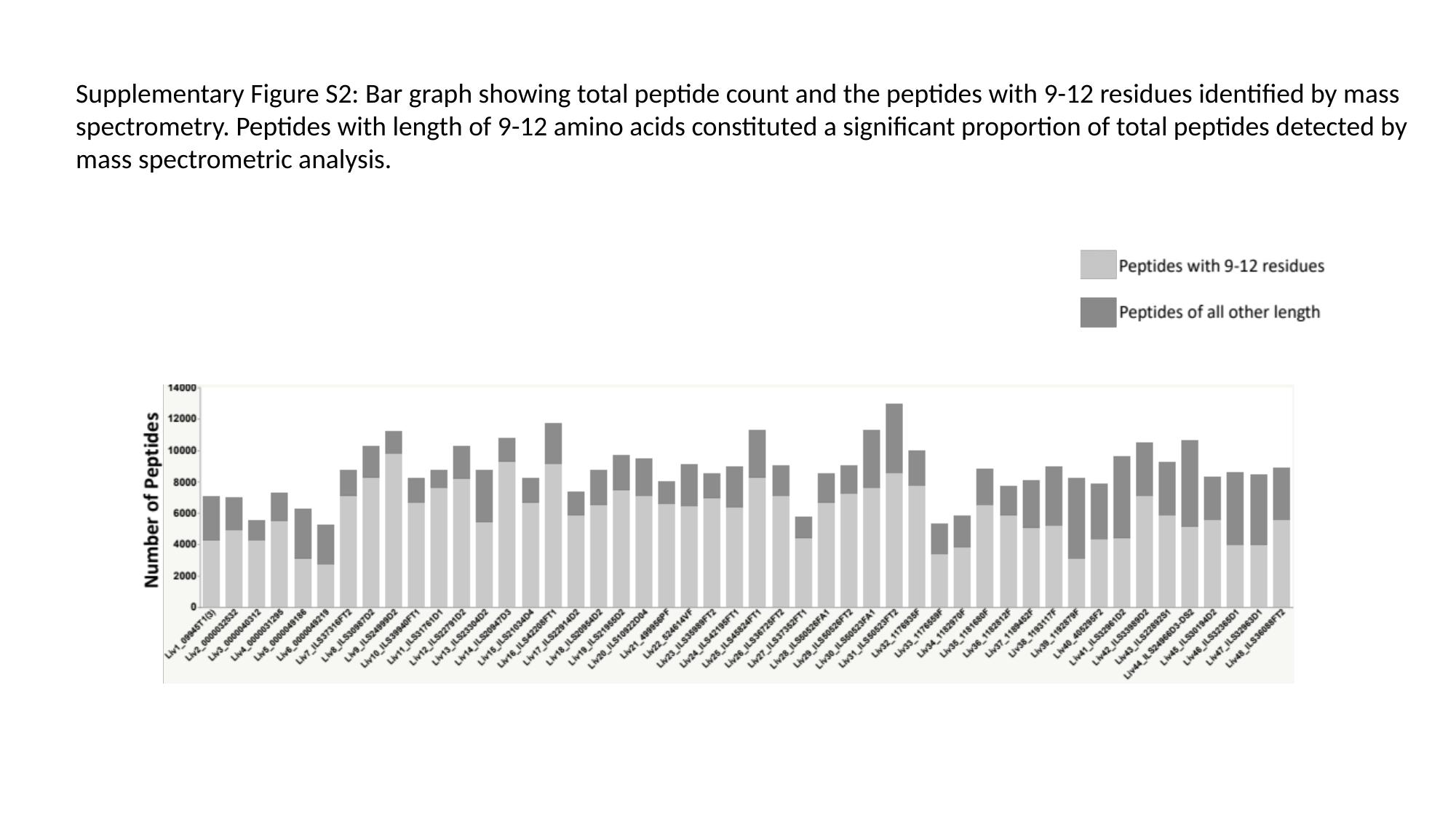

Supplementary Figure S2: Bar graph showing total peptide count and the peptides with 9-12 residues identified by mass spectrometry. Peptides with length of 9-12 amino acids constituted a significant proportion of total peptides detected by mass spectrometric analysis.

## Slide 3
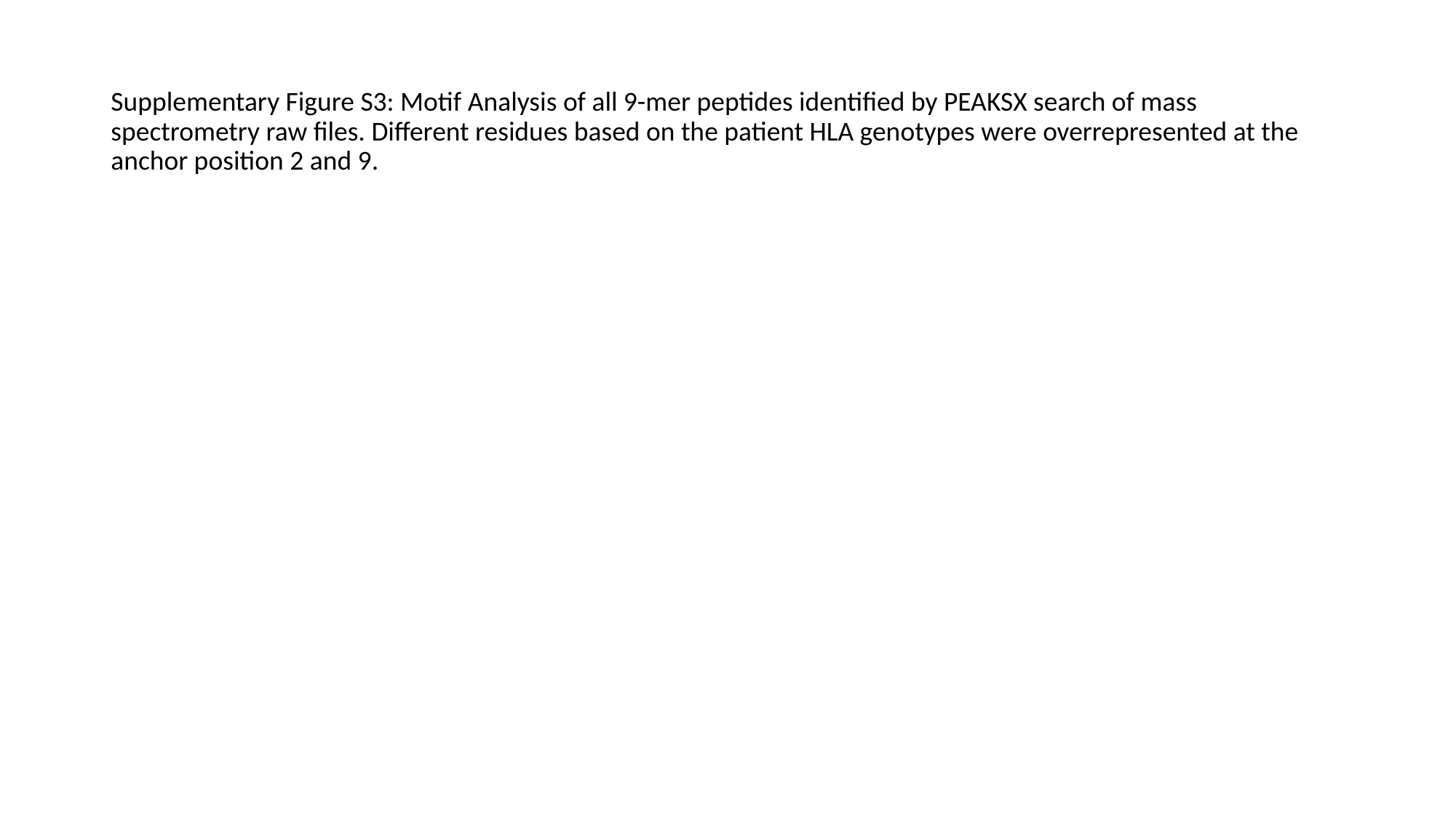

Supplementary Figure S3: Motif Analysis of all 9-mer peptides identified by PEAKSX search of mass spectrometry raw files. Different residues based on the patient HLA genotypes were overrepresented at the anchor position 2 and 9.

## Slide 4
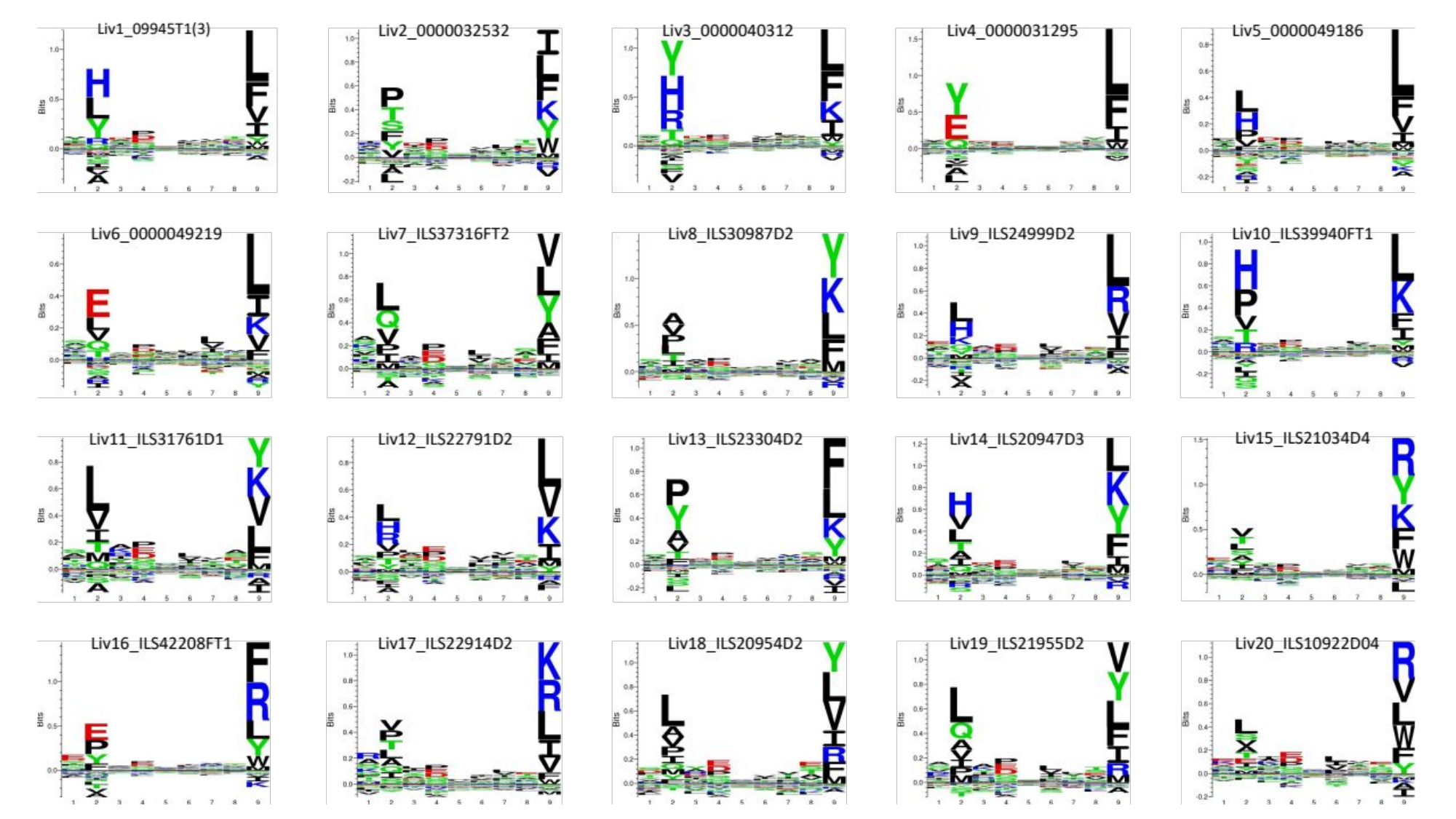

## Slide 5
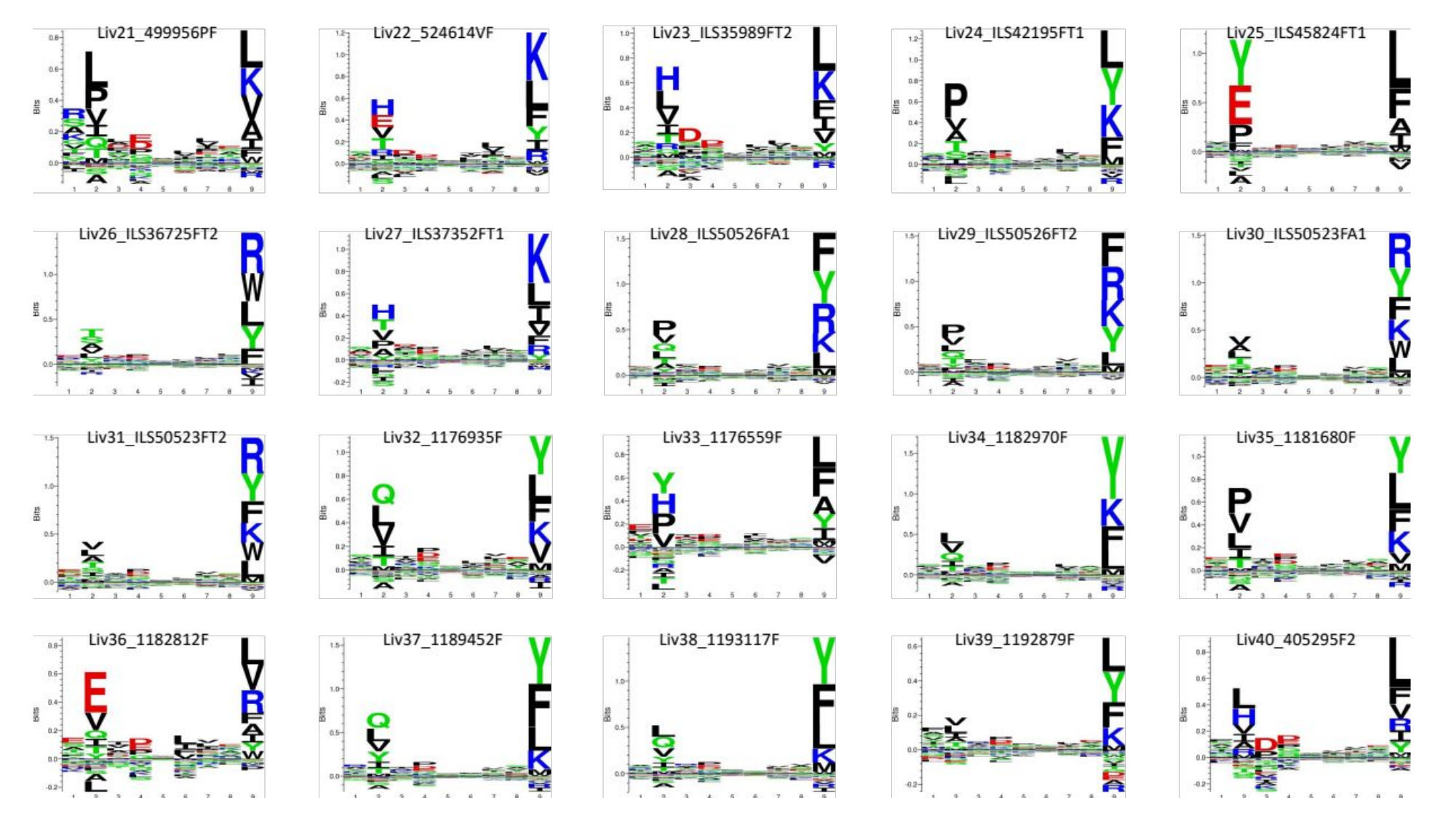

## Slide 6
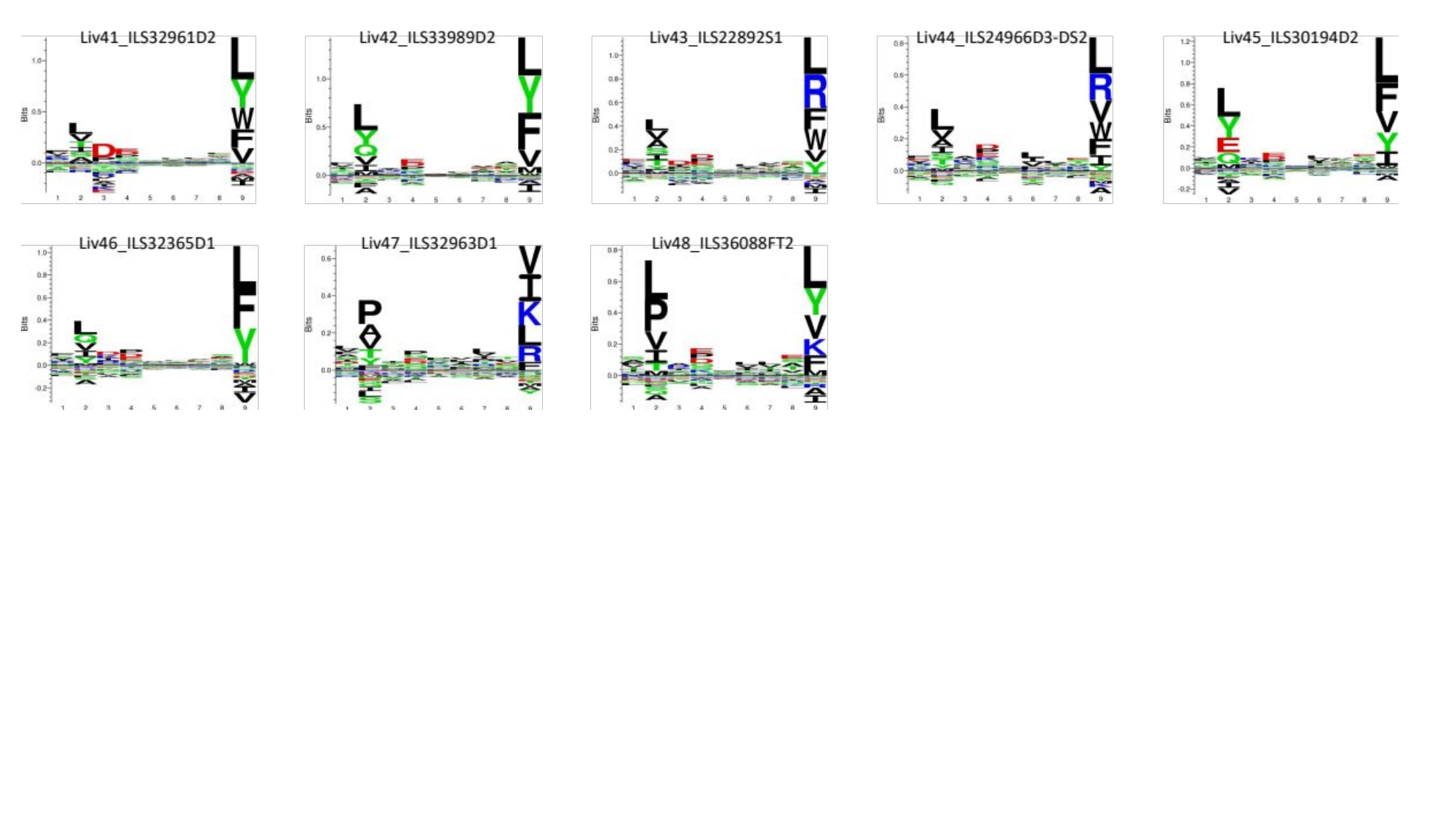

## Slide 7
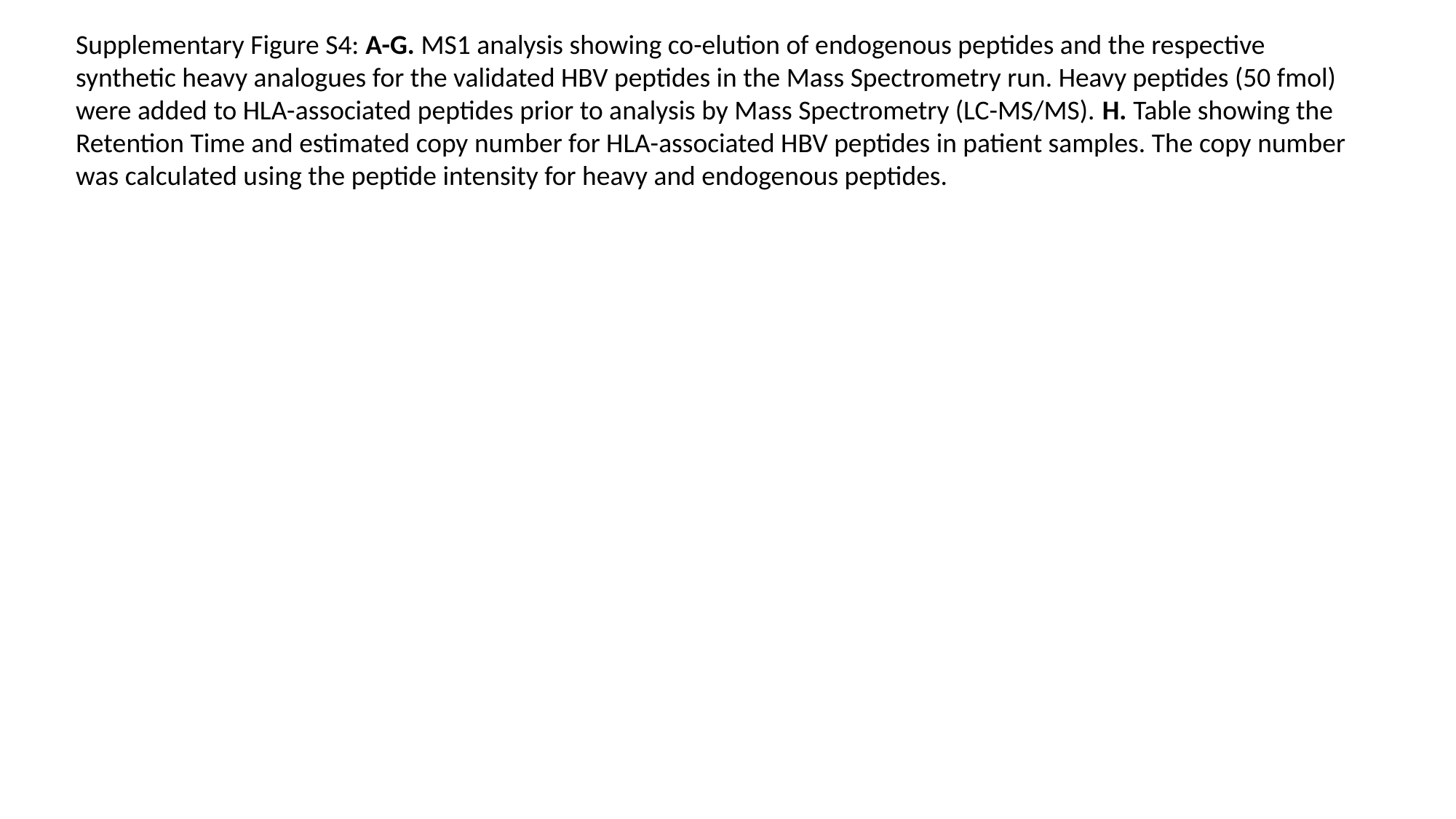

Supplementary Figure S4: A-G. MS1 analysis showing co-elution of endogenous peptides and the respective synthetic heavy analogues for the validated HBV peptides in the Mass Spectrometry run. Heavy peptides (50 fmol) were added to HLA-associated peptides prior to analysis by Mass Spectrometry (LC-MS/MS). H. Table showing the Retention Time and estimated copy number for HLA-associated HBV peptides in patient samples. The copy number was calculated using the peptide intensity for heavy and endogenous peptides.

## Slide 8
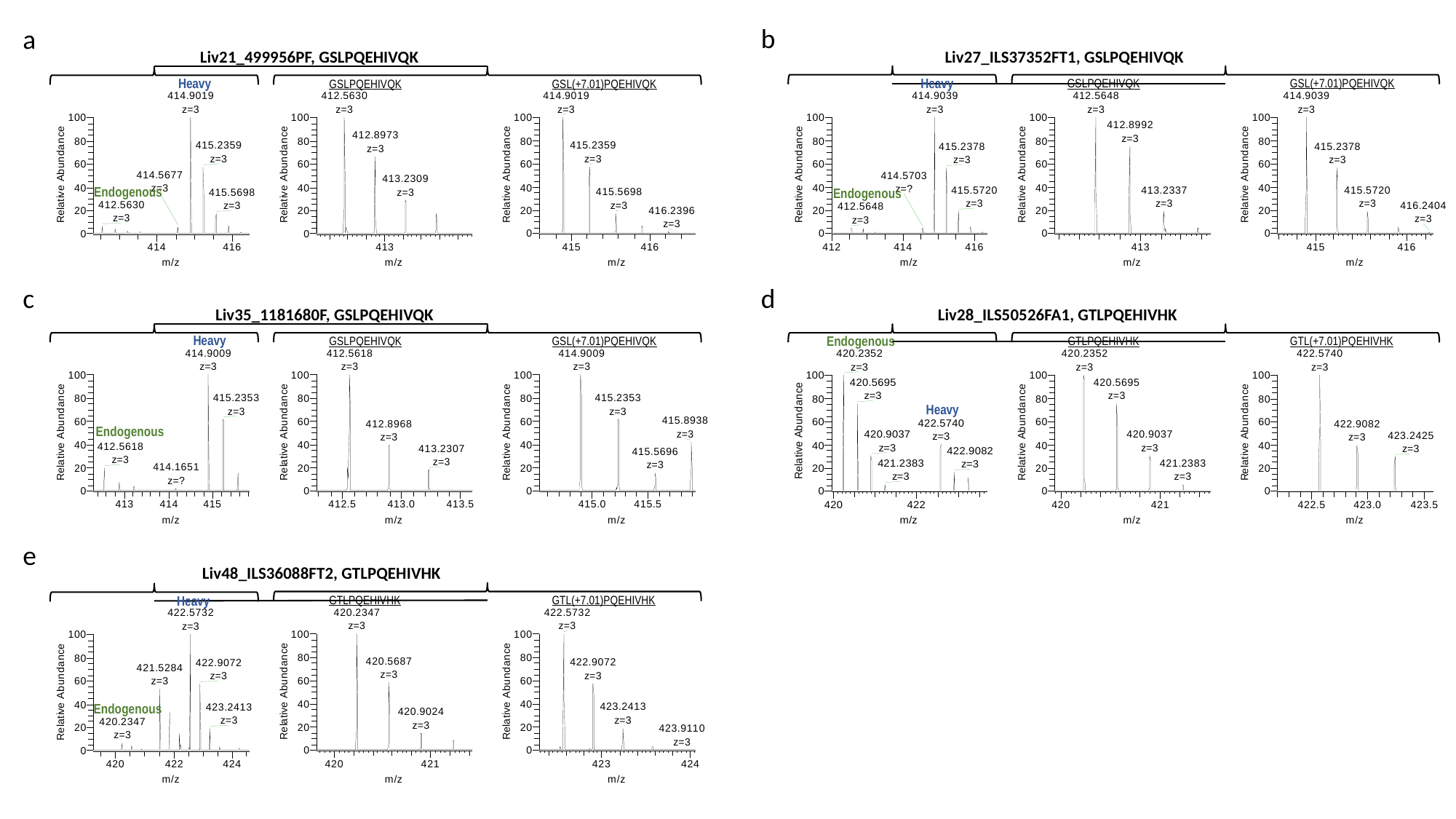

b
a
Liv21_499956PF, GSLPQEHIVQK
Heavy
GSL(+7.01)PQEHIVQK
GSLPQEHIVQK
Endogenous
Liv27_ILS37352FT1, GSLPQEHIVQK
Heavy
GSL(+7.01)PQEHIVQK
GSLPQEHIVQK
Endogenous
c
d
Liv35_1181680F, GSLPQEHIVQK
Heavy
GSLPQEHIVQK
GSL(+7.01)PQEHIVQK
Endogenous
Liv28_ILS50526FA1, GTLPQEHIVHK
Endogenous
GTL(+7.01)PQEHIVHK
GTLPQEHIVHK
Heavy
e
Liv48_ILS36088FT2, GTLPQEHIVHK
GTL(+7.01)PQEHIVHK
GTLPQEHIVHK
Heavy
Endogenous

## Slide 9
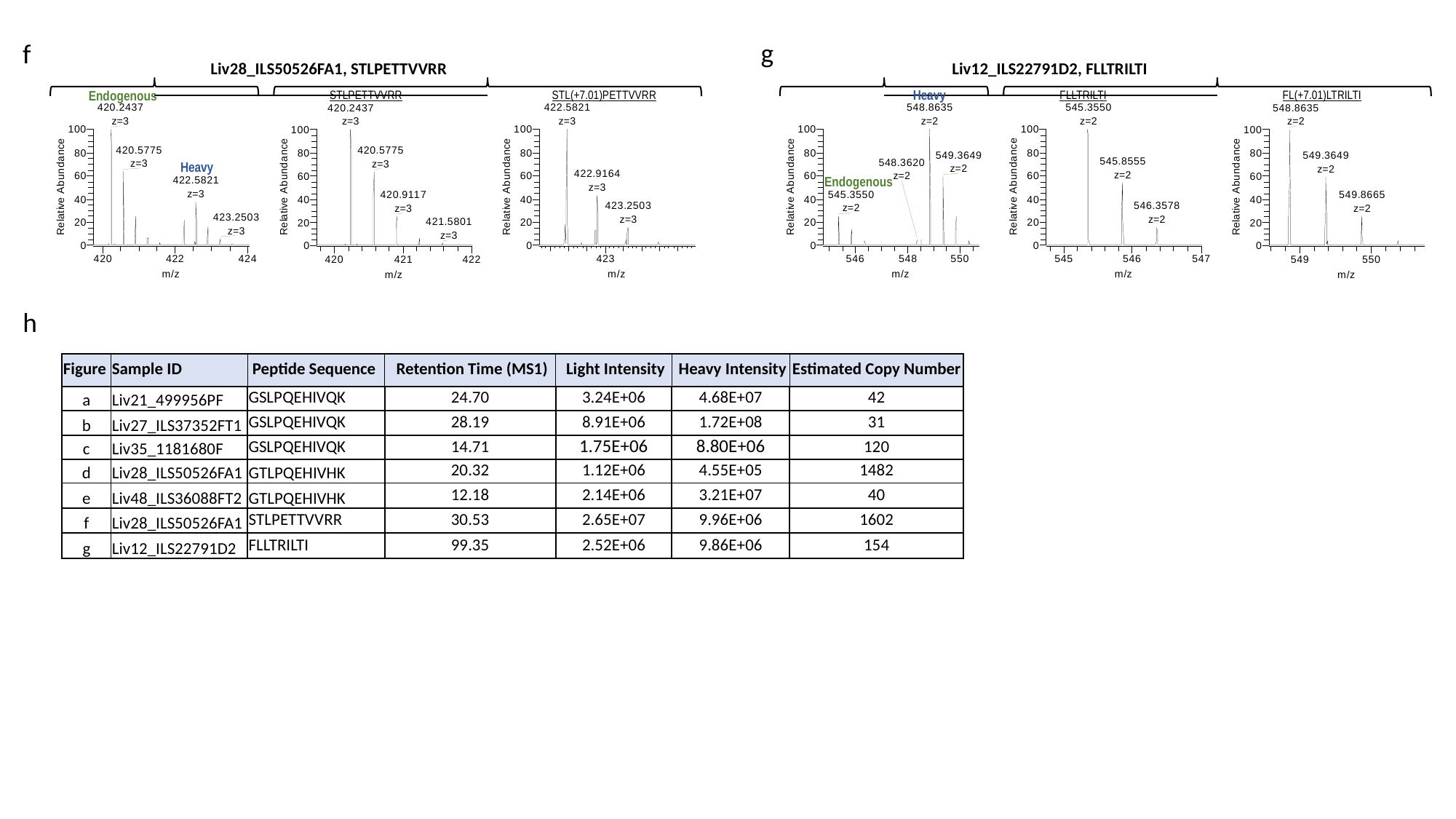

g
f
Liv28_ILS50526FA1, STLPETTVVRR
Liv12_ILS22791D2, FLLTRILTI
Heavy
Endogenous
STL(+7.01)PETTVVRR
FL(+7.01)LTRILTI
STLPETTVVRR
FLLTRILTI
Heavy
Endogenous
h
| Figure | Sample ID | Peptide Sequence | Retention Time (MS1) | Light Intensity | Heavy Intensity | Estimated Copy Number |
| --- | --- | --- | --- | --- | --- | --- |
| a | Liv21\_499956PF | GSLPQEHIVQK | 24.70 | 3.24E+06 | 4.68E+07 | 42 |
| b | Liv27\_ILS37352FT1 | GSLPQEHIVQK | 28.19 | 8.91E+06 | 1.72E+08 | 31 |
| c | Liv35\_1181680F | GSLPQEHIVQK | 14.71 | 1.75E+06 | 8.80E+06 | 120 |
| d | Liv28\_ILS50526FA1 | GTLPQEHIVHK | 20.32 | 1.12E+06 | 4.55E+05 | 1482 |
| e | Liv48\_ILS36088FT2 | GTLPQEHIVHK | 12.18 | 2.14E+06 | 3.21E+07 | 40 |
| f | Liv28\_ILS50526FA1 | STLPETTVVRR | 30.53 | 2.65E+07 | 9.96E+06 | 1602 |
| g | Liv12\_ILS22791D2 | FLLTRILTI | 99.35 | 2.52E+06 | 9.86E+06 | 154 |

## Slide 10
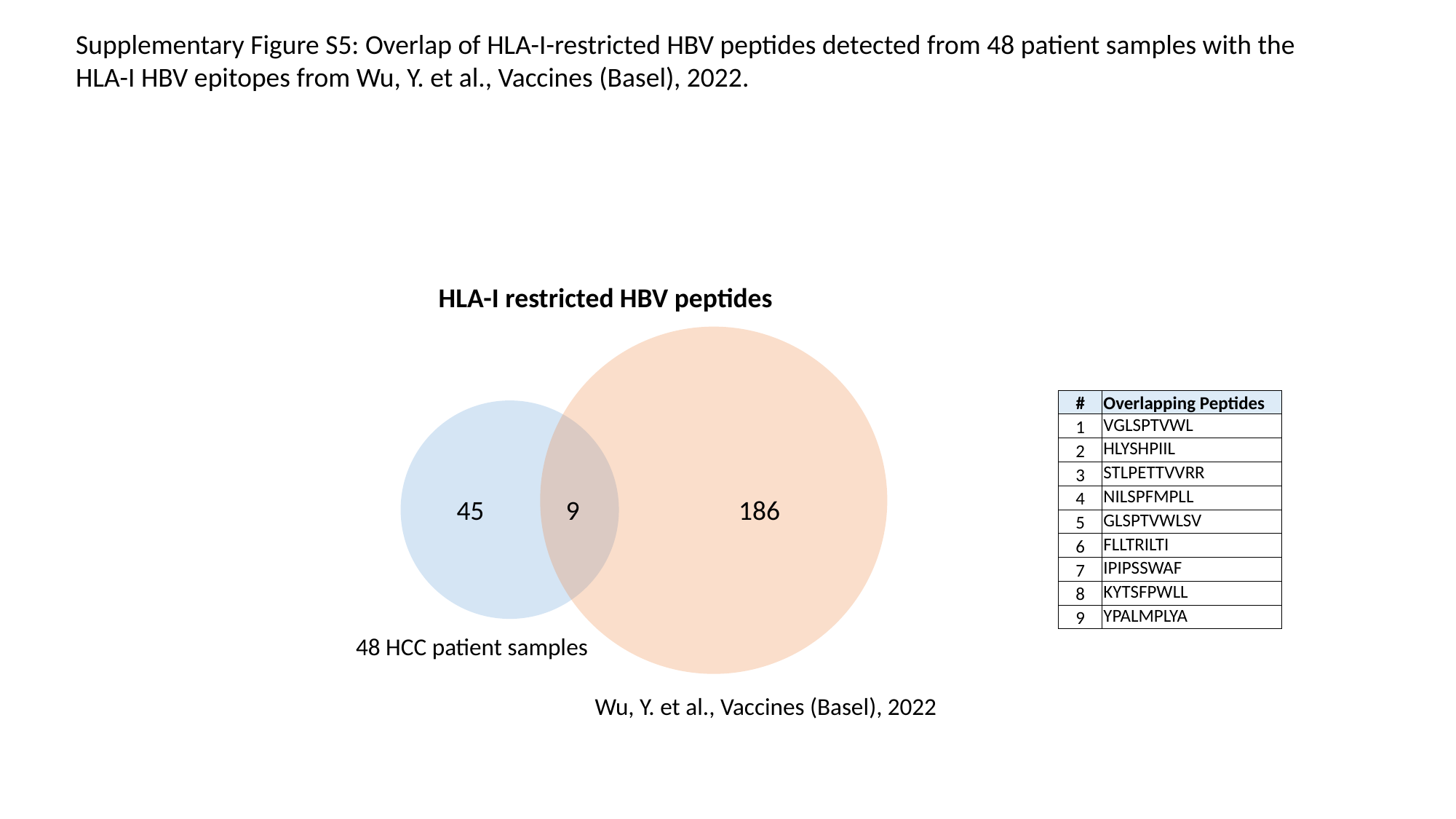

Supplementary Figure S5: Overlap of HLA-I-restricted HBV peptides detected from 48 patient samples with the HLA-I HBV epitopes from Wu, Y. et al., Vaccines (Basel), 2022.
HLA-I restricted HBV peptides
45
9
186
48 HCC patient samples
Wu, Y. et al., Vaccines (Basel), 2022
| # | Overlapping Peptides |
| --- | --- |
| 1 | VGLSPTVWL |
| 2 | HLYSHPIIL |
| 3 | STLPETTVVRR |
| 4 | NILSPFMPLL |
| 5 | GLSPTVWLSV |
| 6 | FLLTRILTI |
| 7 | IPIPSSWAF |
| 8 | KYTSFPWLL |
| 9 | YPALMPLYA |
